# Forecasting Congestion and Enabling Proactive Management on Expressways During Public Holidays: A Survey of Methods, Open Issues, and Research Directions

**DOI:** 10.3390/s26154734

**Published:** 2026-07-26

**Authors:** Zheng Yang, Yizhe Wang, Yan Zhu, Qing Peng

**Affiliations:** 1 School of Naval Architecture Ocean and Civil Engineering, Shanghai Jiao Tong University, Shanghai 200240, China; daredevildd@sjtu.edu.cn; 2 The Key Laboratory of Road and Traffic Engineering, Ministry of Education, Tongji University, 4800 Cao’an Road, Shanghai 201804, China; pengqing_2022@tongji.edu.cn; 3 Jiangsu Runyang Bridge Development Co., Ltd., Bridge Park Shiyezhou, Zhenjiang 212115, China; zhuyan_69@126.com

**Keywords:** holiday traffic congestion, traffic state prediction, proactive traffic control, heterogeneous data fusion, expressway operations management

## Abstract

**Highlights:**

**What are the main findings?**

- Most traffic prediction, congestion identification, and expressway control methods are tuned to regular weekday traffic and transfer poorly to holiday flow marked by short-duration surges and strong spatiotemporal heterogeneity.
- Prediction outputs and control measures remain decoupled, with no closed-loop linkage running from forecasting through warning, control, and feedback.

**What are the implications of the main findings?**

- Building on multi-source heterogeneous data fusion, a closed-loop framework coupling perception, prediction, warning, control, and feedback is proposed to manage the defining characteristics of holiday expressway congestion.
- Priority research directions include holiday-specific non-stationary prediction models, unified congestion-type classification, and integration of reinforcement learning and digital twins for real-time control.

**Abstract:**

Expressway travel demand climbs steeply during public holidays, and the severe congestion that follows has become a principal constraint on both network operating efficiency and the quality of travel services. Dependable forecasting of holiday congestion, coupled with the practical realization of proactive management, accordingly carries considerable theoretical and practical weight for improving how expressways are operated and administered and for safeguarding efficient and safe public travel. Organized around the central theme of congestion prediction and proactive management for expressways over holiday periods, this paper reviews the progress of research across five interrelated areas: traffic speed and flow prediction methods; congestion state identification and forecasting; holiday travel characteristic analysis together with demand prediction; multi-source data fusion and congestion propagation mechanisms; and expressway traffic control together with traveler behavior guidance. Having surveyed the theoretical underpinnings, core technologies, and representative methods of each area, the paper concentrates in particular on the shortcomings and difficulties that existing studies encounter in coping with the hallmark features of holiday traffic, namely demand surges of short duration combined with pronounced spatiotemporal heterogeneity, and proceeds to outline promising avenues for subsequent research. Overall, the review seeks to provide wide-ranging literature support and a theoretical reference for developing technologies that predict and proactively manage congestion on expressways during public holidays.

## 1. Introduction

China’s swift economic and social progress, together with continually rising vehicle ownership, has placed expressways in an ever more central position within the nation’s economy and in people’s everyday mobility. When a major public holiday arrives, however, the volume of expressway travel expands abruptly; trips cluster tightly into particular time bands and particular stretches of road, and the load borne by the network climbs steeply as a result. So familiar has this pattern become that “every holiday brings congestion” has settled into a standing difficulty for road authorities and travelers alike. Beyond the heavy delays and economic costs it imposes, holiday congestion also drives up the likelihood of crashes and raises vehicle emissions, which together strain the travel experience of the public and test the resilience of transportation systems at the regional scale.

Traffic prediction and control have shifted in a fundamental way as the underlying technology has matured: approaches grounded in experience have given way to approaches grounded in data. In a wide-ranging survey of deep learning for traffic prediction, Yin et al. [1] observed that the field has advanced markedly of late. Current methods perform strongly on tasks such as speed prediction, flow prediction, and congestion forecasting. Substantial difficulties remain, however, wherever traffic conditions are dynamic and the spatiotemporal dependencies are intricate and shifting. Concurrently, traffic management and control systems are undergoing fundamental transformation. Wang and Yang [2] reviewed China’s new-generation traffic control technologies and traced the multi-generational evolution of traffic control. They identified three interrelated control paradigms emerging in connected and automated vehicle environments: traffic signal control, connected vehicle guidance, and indirect control of conventional vehicles through connected vehicles. A key finding of their review is that no single mode suffices on its own; systemic performance improvement under heterogeneous traffic, in which connected automated vehicles coexist with human-driven vehicles, requires the synergistic integration of all three paradigms. This insight carries direct implications for expressway congestion management. Urban intersections require coordinated interactions among signals, vehicles, and infrastructure to accommodate diverse vehicle compositions. In the same way, expressways during public holidays demand that variable speed limits, ramp metering, traveler information dissemination, and demand-side guidance be integrated into a unified control framework, because traffic composition and demand patterns vary dramatically over these periods. Against this backdrop, achieving deep integration of advanced prediction technologies with traffic control strategies to construct a congestion prediction and proactive management system tailored to the distinctive characteristics of holiday scenarios has emerged as an important research frontier in intelligent transportation systems.

Holiday traffic, nonetheless, behaves in ways all its own. First, flow swings violently over short time windows, and its spatiotemporal distribution differs markedly from that of an ordinary weekday. Conventional models keyed to historical periodicity are therefore ill-suited to the task. Second, several forces act at once, including abrupt weather shifts, roadway incidents, and diversions prompted by information. The interaction of these forces makes the formation and development of congestion considerably harder to characterize. A good deal has already been accomplished on the theory of traffic prediction, on algorithms for detecting congestion, and on techniques for fusing data; yet reviews and forward-looking assessments aimed specifically at the holiday expressway setting are still comparatively few. Existing surveys have consolidated deep learning methods for general traffic prediction [1], computer vision techniques for intelligent transportation systems, and deep learning applications in congestion detection, prediction, and alleviation, with the latter two surveys examined in Section 3.1. These reviews treat holidays at most as one of several disturbance sources; they do not examine how the defining characteristics of holiday expressway traffic reshape the applicability of prediction, warning, and control methods. The present review differs from them in three respects. It uses the holiday scenario as an explicit analytical lens, assessing for each research area whether the surveyed methods remain valid under short-duration surges, pronounced spatiotemporal heterogeneity, multi-factor coupling, and historical sample scarcity. It connects methodological progress to an operational perspective by organizing the five areas along a perception, prediction, warning, control, and feedback chain. It also foregrounds the institutional context of holiday expressway operations, in particular the toll-exemption policy analyzed in Section 4.1, as a mechanism that distinguishes holiday congestion from ordinary leisure-travel growth.

On this basis, the present paper takes the prediction and proactive management of expressway congestion during public holidays as its core concern and offers a thorough, critically minded account of how the relevant lines of research have progressed. The material that follows is arranged in this way. Section 2 traces how methods for predicting traffic speed and flow have developed, from classical statistical techniques through to deep learning models of space and time. Section 3 turns to the identification and forecasting of congestion states. Section 4 takes up the spatiotemporal signature of holiday traffic, the forecasting of demand, and work specific to expressways. Section 5 considers the fusion of multi-source data and the mechanisms of congestion propagation. Section 6 reviews strategies for controlling expressway traffic and for guiding traveler behavior. Section 7 draws together what has been achieved and where the gaps lie, and sets out directions for future research. Although Section 2, Section 3, Section 5 and Section 6 necessarily draw on the broader traffic engineering literature, each section closes by assessing the surveyed methods against the defining characteristics of holiday traffic, and Section 7 integrates these assessments into a closed-loop framework whose stages correspond one-to-one with the five review dimensions.

**Figure 1:**
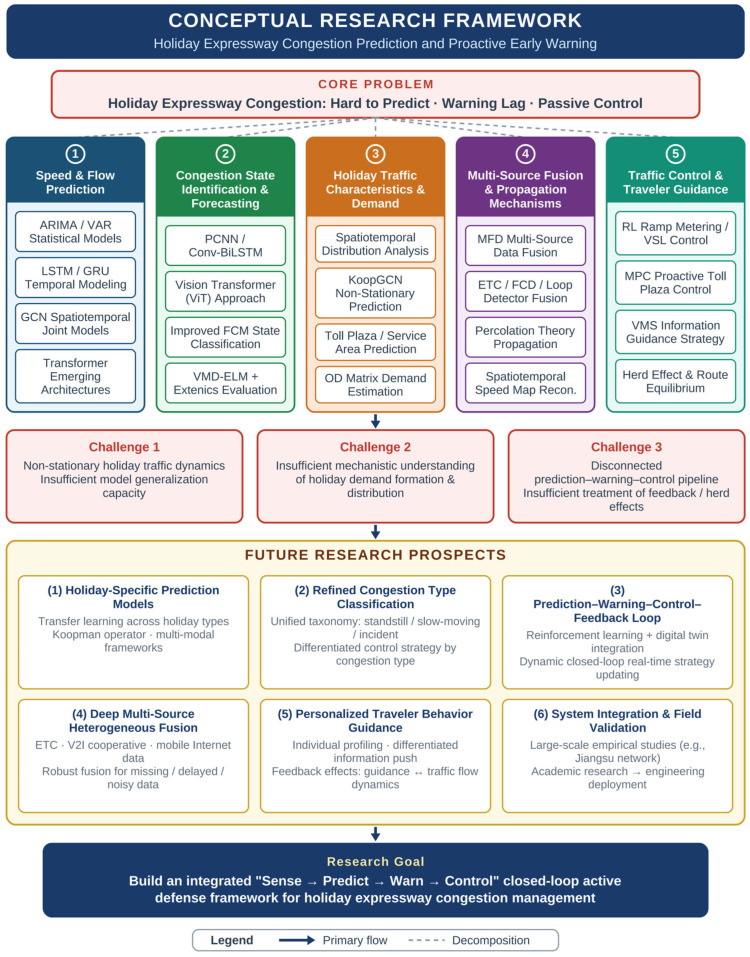
Research framework for congestion prediction and proactive management for expressways during public holidays.

Figure 1 sets out the review’s overall structure, which is built on four tiers: pinpointing the core problem, organizing the work into five research dimensions, distilling the key challenges, and looking ahead to future research.

This paper employs a structured narrative review methodology that incorporates selected systematic screening steps to ensure comprehensive and balanced coverage. The review is narrative in its mode of synthesis, which permits critical, theme-oriented integration across five heterogeneous research areas, while the identification of literature follows the transparent multi-stage procedure described below. Relevant literature was identified through searches of multiple databases. The primary databases include the Web of Science Core Collection, Scopus, IEEE Xplore Digital Library, ScienceDirect, Engineering Village (EI Compendex), the Transportation Research International Documentation (TRID), and China National Knowledge Infrastructure (CNKI), ensuring comprehensive coverage of the interdisciplinary literature spanning transportation engineering, intelligent transportation systems, and computer science.

The search strategy employed combinatorial queries across five thematic domains: (i) traffic prediction terms (“traffic speed prediction,” “traffic flow forecasting,” “short-term prediction,” “deep learning,” “graph neural network,” “Transformer”); (ii) congestion identification and early warning terms (“congestion prediction,” “congestion detection,” “traffic state classification,” “early warning system”); (iii) holiday traffic terms (“holiday traffic,” “festival traffic,” “special event traffic,” “non-recurrent congestion,” “travel demand”); (iv) data fusion and propagation analysis terms (“data fusion,” “macroscopic fundamental diagram,” “congestion propagation,” “percolation theory,” “OD estimation”); and (v) control and guidance terms (“variable speed limit,” “ramp metering,” “variable message sign,” “route choice,” “toll plaza,” “traveler information”). Boolean operators “OR” and “AND” were respectively used within each domain and between domains to construct targeted search expressions. Expressway-specific terms such as “expressway,” “freeway,” “highway,” and “motorway” were applied as additional constraints.

The search timeframe primarily spans from 2010 through early 2026 to cover the complete research trajectory since the introduction of deep learning into traffic prediction. Earlier seminal works prior to 2010 were included only when they held significant value for understanding the evolution of holiday traffic characteristic analysis methods or the theoretical foundations of traveler information guidance.

The literature screening process comprised four stages: (1) preliminary screening based on titles and abstracts to exclude studies clearly unrelated to expressway traffic prediction, congestion control, or holiday traffic analysis; (2) full-text assessment to confirm direct relevance to core themes including traffic speed/flow prediction methods, congestion state identification and early warning, holiday traffic characteristic analysis, multi-source data fusion, and expressway control and traveler guidance; (3) quality assessment, prioritizing peer-reviewed papers published in SCI/SSCI-indexed journals with emphasis on leading transportation journals and high-impact interdisciplinary journals such as Nature Communications; and (4) forward and backward citation tracking to identify important works potentially missed in the initial search.

Inclusion criteria comprised studies directly relevant to traffic prediction, congestion identification, holiday traffic characteristics, data fusion, control strategies, or traveler behavior guidance in expressway scenarios. Exclusion criteria comprised studies lacking relevance to traffic prediction or proactive control, as well as non-peer-reviewed technical reports.

It should be noted that this review does not claim the status of a systematic review in the sense of the PRISMA 2020 statement, and accordingly no formal flow diagram with itemized record counts at each screening stage is reported. The staged procedure described above is intended to make the search logic transparent and repeatable, while the final corpus was assembled purposively, prioritizing methodological representativeness and thematic relevance to the holiday expressway setting over exhaustive enumeration. The limitations that follow from this choice are acknowledged in Section 7.1.

Of the 71 publications ultimately included in this review, 50 were published in 2020 or later, accounting for approximately 70%, reflecting the rapid advancement of deep learning and intelligent transportation technologies in recent years. The remaining 21 publications from 2019 and earlier primarily cover early statistical analyses of holiday traffic characteristics, classical empirical studies on the influence of traffic information on traveler behavior, and foundational methodological works in traffic prediction and control, reflecting this review’s thorough coverage of core literature in the transportation engineering domain.

In terms of geographic coverage, the corpus combines a substantial body of China-based studies with international evidence, including studies grounded in data from the United States, Canada, Australia, Switzerland, Turkey, Japan, Spain, and The Netherlands, together with multi-city empirical analyses. At the same time, the corpus deliberately emphasizes Chinese expressway settings, and this emphasis reflects the subject itself rather than convenience of access. The combination of concentrated statutory holidays, a very large national expressway network, and a toll-exemption policy for eligible passenger cars during major public holidays produces holiday congestion whose intensity and formation mechanism are documented most extensively in the Chinese literature. Section 4.1 examines this institutional mechanism in detail, and Section 7.1 states the corresponding limitation on the transferability of the surveyed findings to regions with different holiday structures and toll policies.

## 2. Methods for Predicting Traffic Speed and Flow

At the heart of any congestion early warning system lies the accurate prediction of traffic speed and flow. Speed is the most immediate indicator of how a road is operating, mirroring directly the degree of congestion and the level of service, which is why prediction built around speed occupies such a prominent place in expressway warning applications. The methods themselves have moved over recent years from statistical formulations, through machine learning, and on to deep learning, with each step enlarging the model’s ability to capture the spatiotemporal behavior of traffic flow. The review in this section is organized under four technical headings: statistical and traditional machine learning methods; temporal modeling based on recurrent neural networks; joint spatiotemporal modeling based on graph neural networks; and Transformer architectures along with other emerging methods.

### 2.1. Statistical and Classical Machine Learning Approaches

The early stage of traffic prediction research primarily relied on classical time series analysis methods. Statistical approaches such as AutoRegressive Integrated Moving Average (ARIMA) models, Vector AutoRegression (VAR) models, and spatiotemporal (ST) models achieved short-term prediction through modeling the linear autocorrelation structures in historical traffic data. As an early attempt to move beyond such linear formulations, Yu et al. [3] proposed the spatiotemporal recurrent convolutional network (SRCN), which represents the road network as a sequence of network-wide traffic speed images and combines deep convolutional neural networks (DCNNs) with long short-term memory (LSTM) networks so that the DCNN captures spatial dependencies across the network while the LSTM learns the temporal dynamics. Experiments on a Beijing transportation network with 278 links indicated that the SRCN outperformed other deep learning-based algorithms in both short-term and long-term traffic prediction. This early effort reflects the recognition that data-driven architectures with greater representational capacity could complement and extend traditional statistical approaches in modeling complex traffic dynamics.

In a comparable vein, Chen et al. [4] worked from the standpoint of decomposing traffic flow, separating it into a periodic part and a fluctuating part; the periodic part was described with Fourier transforms, while the spatiotemporally correlated fluctuating part was modeled through supervised learning. Tested on electronic vehicle identification data, the resulting TSA-SL hybrid scheme outperformed conventional methods that treat the flow as a whole. What such work shows is that competitive accuracy is within reach even for traditional methods, provided the essential structure of traffic flow is probed in depth and decomposed in a suitable way.

The basic weakness of statistical models, however, is that they lean too heavily on assumptions of linearity. Once traffic passes through the strongly nonlinear transitions between states, from free flow into congestion and back toward recovery, the accuracy of these traditional methods drops off sharply. It is this limitation that has pushed researchers toward deep learning, where the representational capacity is far greater.

### 2.2. Recurrent Neural Network-Based Temporal Prediction

Traffic prediction was transformed once deep learning entered the picture. Long Short-Term Memory (LSTM) networks, able as they are to curb the vanishing gradient problem through gated memory and to pick up long-range temporal dependencies on their own, have come to serve as the foundational architecture for predicting traffic time series. In early landmark studies, LSTM rapidly established its benchmark status in traffic speed prediction owing to its accuracy and stability advantages. Concurrently, the application scope of deep learning expanded from pure speed prediction to broader traffic flow prediction. Combining L1-regularized linear layers with stacked tanh nonlinear layers, Polson and Sokolov [5] built a deep learning architecture and, using sensor data from Chicago’s I-55, showed that it could predict short-term flow accurately even amid the sharp swings brought on by special events such as American football games and severe snowstorms, a result that offers a useful methodological template for modeling under special event conditions. Hsueh and Yang [6] brought vehicle type composition and the speed variation in upstream segments into the LSTM framework as additional features, and in doing so brought to light how the correlation between vehicle speed and the distribution of vehicle types helps lift prediction accuracy.

It is noteworthy that recurrent architectures face inherent limitations when extended to network-scale traffic prediction. Zhang et al. [7], through a systematic empirical comparison of LSTM against twelve advanced spatiotemporal forecasting models on five large-scale benchmark datasets, demonstrated that LSTM, despite its strength in capturing short-term and long-term temporal dependencies, exhibits markedly weaker performance than methods that explicitly model the road network as a graph and characterize heterogeneous traffic patterns across spatial regions. The study further indicated that traffic flow data exhibit pronounced heterogeneity across both temporal scales and spatial regions, with periodic patterns varying substantially across days and fluctuation intensities differing among sensors within the same network, characteristics that recurrent architectures with shared parameters and purely sequential processing have difficulty representing. These findings reveal a fundamental gap between the temporal-only modeling capacity of LSTM-type architectures and the joint spatiotemporal modeling demands of real-world road networks, motivating the architectural shift toward graph-based methods discussed in the next subsection.

On the side of architectural refinement, Zheng et al. [8] folded attention into the Conv-LSTM framework so that the importance of flow sequences at different time steps could be weighed automatically, sharpening short-term prediction; bidirectional LSTM was used in parallel to draw out daily and weekly periodic features, so that both long-term and short-term temporal patterns were captured together. Abduljabbar et al. [9] systematically compared the prediction performance of unidirectional and bidirectional LSTM on multiple Australian expressway datasets, finding that a four-layer stacked BiLSTM network can maintain above 90% accuracy in speed prediction within a 60-min prediction horizon, though flow prediction accuracy decays more noticeably with horizon extension, a comparative conclusion that provides quantitative reference for LSTM architecture selection in engineering practice.

Taken together, the progression from unidirectional LSTM to bidirectional and stacked designs shows that recurrent networks can markedly strengthen prediction by enriching the directions of information flow and deepening the network. Their grasp of spatial correlation structure, even so, remains limited. As the scope of prediction widens from a single detector to the whole network, temporal modeling on its own cannot adequately convey the topological links between road segments or the way disturbances propagate across space, and it is this basic shortfall that has prompted the broad adoption of graph neural networks in traffic prediction.

### 2.3. Spatiotemporal Modeling with Graph Neural Networks

Among the more consequential methodological advances of recent years in traffic prediction is the practice of representing the road network explicitly as a graph and drawing out spatial features through graph convolution. Within this line of work, pairing Graph Convolutional Networks (GCN) with recurrent networks has emerged as the mainstream approach. Yu et al. [10] refined GCN so that differing connection strengths among neighboring segments could be told apart, in contrast to the uniform weighting of neighbors in a standard GCN, and added a generative adversarial framework to keep the predicted probability distribution consistent with the joint density of the actual traffic states. For prediction from GPS trajectory data, Bogaerts et al. [11] joined Graph CNN to LSTM and devised a dimensionality reduction technique grounded in temporal correlation that picks out the most informative segments to feed the model; on DiDi ride-hailing data from Xi’an and Chengdu in China, the approach held up steadily across horizons stretching from 5 min to 4 h.

In terms of scalability for large-scale road networks, Zhang et al. [12] modeled network traffic dynamics as a Markov chain on directed graphs and proposed the Spatiotemporal Graph Inception Residual Network (STGI-ResNet), which effectively extracts multi-periodic spatiotemporal features while maintaining linear computational complexity relative to the number of road segments through the fusion of residual learning and Inception structures, outperforming multiple baseline methods in multi-step predictions from 10 to 60 min on large-scale networks. Huang et al. [13] proposed the multi-scale temporal dual graph convolution network (MD-GCN) to address the joint extraction of temporal features and spatial node correlations in traffic flow. The model employs a gated temporal convolution built on a channel attention mechanism and an inception structure to extract multi-scale temporal dependencies, and develops a dual graph convolution module in which a graph sampling and aggregation submodule (GraphSAGE) captures local correlations between neighboring nodes while a mix-hop propagation graph convolution submodule (MGCN) captures global correlations. Experiments on multiple public traffic datasets demonstrated that the model outperforms existing methods in traffic flow forecasting.

The introduction of attention mechanisms has further enhanced the expressive capacity of spatiotemporal models. The Graph Attention Temporal Convolutional Network (GATCN) of Zhang et al. [14] leans on graph attention networks to uncover intricate spatial correlations across the road network, on temporal convolution to register temporal dependencies, and on multi-head self-attention to extract the coupling between space and time. By reading the graph attention heat maps, the experiments were able to tell the distinct traffic behaviors of expressway ramps and mainlines apart. For the more challenging task of multi-step prediction, Zhang et al. [15] proposed the Attention Graph Convolution Sequence-to-Sequence model (AGC-Seq2Seq), which uses the Seq2Seq architecture and graph convolution to, respectively, model temporal and spatial dependencies, and designs an attention-based training method to overcome error accumulation in multi-step prediction, with experiments revealing the dynamic variation in traffic spatiotemporal correlation with prediction horizon and segment location.

The above studies collectively demonstrate that the development of graph neural networks is advancing along two complementary directions: first, improving graph convolution operations (e.g., heterogeneous weights, K-order neighborhoods, attention mechanisms) to enhance the expressive capacity of spatial features; and second, integrating external attributes and multi-step prediction architectures to strengthen model adaptability to complex real-world scenarios. Table 1 provides a comparative summary of the core architectural features of these representative spatiotemporal prediction models.

### 2.4. Transformer-Based and Other Emerging Approaches

For all that has been gained by combining graph neural networks with recurrent networks, the inherently serial computation of recursive designs such as LSTM caps training efficiency and still suffers information decay when sequences grow very long. The Transformer architecture, resting on the globally parallel computation that self-attention permits, opens a fresh route past this bottleneck. Zhang et al. [16] applied the Temporal Fusion Transformer (TFT) to expressway speed prediction, using multi-head attention to capture short-term and long-term temporal dependencies at once and a fusion decoder to bring together inputs of several types; on Minnesota expressway data, TFT proved especially advantageous for medium- to long-term horizons of 30 min and longer.

Yan et al. [17] further proposed the “Traffic Transformer” algorithm, designing global encoder and global–local decoder components to, respectively, extract and fuse spatial patterns at global and local scales, and leveraging the stacked layer structure of multi-head attention mechanisms to learn dynamic and hierarchical spatiotemporal features of traffic flow. That study identified three overlooked spatial attribute issues in traditional methods, specifically that traffic flow propagation does not follow network topology, spatial patterns vary over time, and a single adjacency matrix is inadequate for complex hierarchical urban traffic flow, while noting that the Transformer’s global attention mechanism provides a natural framework for addressing these issues.

Pushing prediction to a finer grain, Tang et al. [18] moved the scale from the usual segment level down to the lane level, putting forward a three-dimensional dual-attention convolutional model to capture high-dimensional spatiotemporal features spanning segments, lanes, and time periods; on Shanghai loop detector data it reached a mean absolute error of 2.9 km/h and laid bare how much speed can vary from lane to lane within one and the same segment. Shabarek et al. [19], in addition, assembled a deep learning framework attuned to the effects of adverse weather, feeding real-time meteorological variables such as wind speed, rainfall intensity, and visibility into the prediction models and thereby laying a technical foundation for coping with the traffic swings that abrupt weather shifts, including those that can arise over holidays, are apt to produce. In the data acquisition dimension, traditional fixed detectors are constrained by inherent limitations such as limited spatial coverage and high maintenance costs, particularly acute on expressway segments lacking detection equipment. To address this, Wu et al. [20] proposed an anisotropic Gaussian process method for freeway traffic state estimation from vehicle trajectory data, introducing a kernel rotation reparametrization scheme that explicitly models the directional propagation of congestion waves while providing statistical uncertainty quantification for the imputed states. Validation across the NGSIM and HighD freeway trajectory datasets under varying connected vehicle penetration rates demonstrated state-of-the-art accuracy in scenarios with sparse observations, indicating that vehicle trajectory information can serve as a spatially continuous data source that effectively compensates for the inherent coverage limitations of fixed-sensor infrastructure on freeway corridors.

Across the whole arc from statistical methods to deep learning models of space and time, both the accuracy and the reach of prediction have improved by leaps. Most of the methods surveyed above, however, were devised and tested against the traffic patterns of ordinary weekdays, and how well they generalize and how quickly they respond when faced with the strong non-stationarity, abruptness, and heterogeneity of holiday flow has yet to be established. Viewed more closely through the holiday lens, the four method families surveyed in this section fall short in distinguishable ways. Statistical and classical machine learning models depend on stable periodicity and are structurally unable to represent the regime shifts that concentrated departures and toll-free windows (Section 4.1) create. Recurrent architectures can in principle learn holiday patterns, but the scarcity of holiday samples, a handful of comparable periods per year, starves them of training data precisely where flexibility is most needed. Graph-based spatiotemporal models capture propagation along the network, yet their adjacency structures and learned weights are typically calibrated on weekday flows, so the spatial correlation patterns they encode may no longer hold once the origin-destination structure rotates toward intercity and tourist corridors. Transformer-based models offer the strongest capacity for long-range and non-stationary dependencies, and evidence from special events and non-stationary expressway flow is encouraging, but systematic benchmarking on holiday expressway data remains absent. The binding constraint, in short, is not model capacity but the mismatch between the conditions the models are trained on and the conditions holidays present. More to the point, predicting speed or flow accurately is only the opening move in congestion warning. Turning those predictions into dependable identification of congestion states and into graded warnings is the crucial bridge between prediction technology and management decisions, and it is exactly this that the next section takes up.

## 3. Identification and Forecasting of Traffic Congestion States

Where the prediction of speed and flow supplies the quantitative groundwork for congestion warning, the accurate identification and forecasting of congestion states is the step that converts a stream of predicted values into decisions a manager can act on. Identifying congestion means settling not merely whether it is present, but a series of harder questions in turn: how severe it is, what kind of congestion it is, and how it will unfold. The review in this section proceeds on three levels: the modeling of congestion prediction, the classification and identification of states, and integrated early warning systems.

### 3.1. Deep Learning Models for Congestion Prediction

Closely related though it is to speed and flow prediction, congestion prediction differs from it at root, for it is usually cast as a classification problem, with traffic sorted into congested, non-congested, or transitional, and it must capture the telltale features of the transitions between states. From a methodological review perspective, Dilek and Dener [21] surveyed computer vision applications in intelligent transportation systems, examining the machine learning and deep learning methods applied to problems such as traffic monitoring and control, incident detection and management, road usage pricing, and road condition monitoring. Drawing on more than 300 studies, the survey assessed the applicability of these techniques together with the advantages they offer and the difficulties they present, and outlined future research areas and trends for improving the efficiency and safety of intelligent transportation systems. Kumar and Raubal [22] systematically reviewed the application of deep learning in three task categories, namely congestion detection, prediction, and alleviation, from the perspective of distinguishing between recurrent and non-recurrent congestion, revealing inherent challenges and gaps in current research and providing important guidance for future research directions.

Chen et al. [23] built the PCNN method on two empirical findings, namely that congestion patterns resemble one another across adjacent time periods and across successive working days, and that the severity of congestion is multi-scale in nature; by folding the time series into two-dimensional matrices fed to deep convolutional networks, the method learns at multiple granularities, registering the broad trend of congestion at the macroscopic scale while picking up its detail at the microscopic scale. Taking the microscopic view of how vehicular disturbances spread, Elfar et al. [24] drew on the vehicle trajectory data that connected vehicle environments make available and used logistic regression, random forest, and neural network methods to examine how disturbances of individual vehicles relate to the formation of shockwaves; offline and online models were both constructed, and congestion state prediction reached an accuracy as high as 97%.

Working on joint spatiotemporal congestion prediction, Li et al. [25] put forward the Conv-BiLSTM module, which first draws spatial features out of speed data with a CNN and then captures temporal and alignment features with a bidirectional LSTM; on Shanghai expressway data, the predicted congestion states agreed closely with the actual ones. Ramana et al. [26] carried the Vision Transformer (ViT) into congestion prediction, passing CNN-generated feature maps through the tokenization and projection mechanisms of ViT before handing them to an LSTM network; under anomalous traffic conditions, the method clearly outperformed traditional ones, marking an early foray of Transformer architectures into congestion forecasting. Notably, the evolution of traffic states is also profoundly influenced by external factors such as weather. Braz et al. [27] evaluated several deep learning methods, including long short-term memory, autoregressive LSTM, and a convolutional neural network, to forecast road traffic flow from radar and meteorological sensor data in a coastal resort area where traffic depends strongly on weather conditions. Their results indicate that meteorological variables were essential for the forecast and that reasonable accuracy could be achieved for one-hour-ahead prediction, with the convolutional neural network yielding the lowest prediction error and the shortest computation time. This study illustrates that incorporating environmental factors into prediction frameworks constitutes a critical pathway for improving reliability under adverse weather conditions, a point of particular importance for sudden congestion triggered by abrupt weather changes during holiday periods.

### 3.2. Methods for Classifying Traffic States

Before differentiated control strategies can be put in place, congestion states must be classified with precision. Distinct kinds of congestion, a localized standstill as against slow-moving heavy traffic, for instance, differ fundamentally in how they propagate, how far they reach, and how best to respond to them, and so call for different methods of identification and different management approaches.

From the angle of building a comprehensive indicator system, Cheng et al. [28] introduced a new classification metric, the “ample degree of road network,” and combined it with the conventional parameters of flow, speed, and occupancy. With a Fuzzy C-Means (FCM) clustering method improved in both its membership function and its sample weighting, they classified traffic states on a Shanghai urban expressway. Set against conventional FCM, SVM, decision tree, and KNN methods, the improved FCM raised overall classification accuracy by 10.10%, 5.45%, 30.92%, and 35.66%, respectively. Concentrating on a thorough mining of expressway speed features, Zhao et al. [29] proposed a traffic state identification model built on the Multi-view Fusion Chebyshev Graph Convolutional Network (MvFCGCN), fusing weighted checkpoint data with radar speed data, rendering them as heat map feature images, and then labeling traffic states automatically by means of deep clustering networks, for an overall identification accuracy of 95.25% in the end. Worth noting is that the complementary multi-source fusion strategy employed here carries real reference value for the difficulty of scarce data sources in expressway settings.

### 3.3. Comprehensive Systems for Congestion Early Warning

Converting prediction results into dependable congestion-level warnings is a pivotal step on the road to proactive traffic management. Jiang et al. [30] assembled a traffic congestion early warning system out of four modules: point prediction, feature estimation, interval prediction, and comprehensive evaluation. In the point prediction stage, variational mode decomposition is fused with improved extreme learning machines to predict traffic parameters precisely, with MAPE values of 3.63%, 3.72%, and 4.51% for segment density, occupancy, and average speed, while the comprehensive evaluation stage brings in extension theory to grade congestion, arriving at congestion-level prediction accuracy above 97%. What makes the system valuable is the way a systematic evaluation framework turns continuous predicted parameter values into discrete congestion-level judgments, closing the distance from prediction to early warning.

Table 2 lays out a systematic comparison of these congestion identification and early warning methods along four axes: method category, core technology, identification target, and key contribution. The table makes plain a clear trajectory, from prediction alone toward a full pipeline that joins prediction, classification, and early warning: the earlier methods, PCNN and Conv-BiLSTM among them, dwelt on binary or multi-class prediction of congestion states; mid-stage work such as improved FCM and MvFCGCN moved toward a finer grading of congestion levels; and recent efforts, exemplified by the extension-theory warning system, have set out to link the entire chain from parameter prediction through to graded warning. It is worth noting that validation has rested mainly on regular traffic data, and the case for applicability to the irregular patterns of holiday traffic remains comparatively thin.

Two deeper issues deserve emphasis beyond this trajectory. First, nearly all identification methods reviewed here define congestion relative to a recurrent baseline, whether through clustering on conventional flow, speed, and occupancy parameters [28] or through learning from patterns shared across successive working days [23]. Under holiday conditions, the baseline itself shifts: a state that would count as severe congestion on an ordinary Tuesday may be the expected operating point of a holiday afternoon, so thresholds calibrated on weekday data risk both over-alarming and under-alarming. Second, the accuracy figures collected in Table 2, frequently above 95%, are obtained on datasets dominated by recurrent congestion. They should not be read as evidence of readiness for holiday deployment, because surge-driven, non-recurrent congestion is precisely the regime in which such classifiers are least tested [22]. A management-oriented classification standard would therefore need to be conditioned on calendar context rather than defined in absolute terms.

To sum up, techniques for identifying and forecasting congestion states have grown from simple threshold-based discrimination into a comprehensive system that weaves together deep learning prediction, multidimensional feature classification, and systematic evaluation. Several gaps nonetheless persist. Most methods are built around regular traffic patterns and pay too little heed to the heterogeneity of holiday traffic; standards for classifying congestion types have yet to be unified, and systematic classification frameworks geared to management decisions are lacking; and the path from prediction to early warning remains less than smooth, above all in how differentiated responses should be triggered for different types of congestion. These shortcomings stand out all the more in the holiday setting, whose intrinsic traffic characteristics and dedicated prediction methods the next section examines in detail.

## 4. Analysis of Holiday Traffic Characteristics and Demand Forecasting

What sets holiday traffic apart is how far it departs from the regular pattern: the spatiotemporal distribution of demand, the mix of trip purposes, the proportions of vehicle types, and the characteristics of driving behavior all shift appreciably, with the upshot that models trained on everyday conditions tend to fare poorly in holiday scenarios. This section looks first at progress on the spatiotemporal characteristics of holiday traffic, then at congestion prediction and demand analysis methods particular to expressways, and finally at OD estimation techniques within demand prediction.

### 4.1. Spatial and Temporal Patterns of Holiday Traffic

An effective prediction model presupposes a deep understanding of how holiday traffic behaves. Back in 2010, Jun [31] looked into how speed distributions vary on interstate highways over the Thanksgiving holiday, using Gaussian mixture speed distribution models and the EM algorithm to bring out ways of quantitatively characterizing the severity and variability of holiday congestion. In still earlier work, Liu and Sharma [32] drew on twenty years of permanent traffic counter records from Alberta, Canada, and tested in systematic fashion the significance of holiday effects on weekly, daily, and hourly volumes by means of the non-parametric Wilcoxon signed-rank test and Friedman methods; they found that holidays contribute substantially to the variability of flow and that directional holiday peaks are pronounced. Cools et al. [33] weighed how well different modeling approaches explain and predict daily holiday volumes, and found the Box–Tiao approach to outdo the alternatives plainly, both in quantifying holiday effects and in long-horizon forecasting.

At the microscopic level of holiday travel behavior, Yang et al. [34] compared holiday and working-day differences in travel mode and trip chain choices based on Beijing travel survey data, finding that the decision structures between the two are diametrically opposite: working-day travelers tend to first determine the trip chain pattern before selecting the travel mode, whereas holiday travelers first select their travel mode and then organize the trip chain. More importantly, holiday travel exhibits stronger car dependency, and travelers are more sensitive to time changes than to cost changes. These behavioral characteristic differences reveal, from a micro-mechanistic perspective, the deep-seated reasons why holiday traffic demand differs from the everyday. From the perspective of congestion mechanism modeling, Bao et al. [35] employed bottleneck models to characterize the evolution of traffic congestion from residential areas to tourist destinations during holidays, focusing on the impact of China’s expressway holiday toll exemption policy on travelers’ departure time choices and social welfare. They obtained analytical solutions for departure time variations and social efficiency losses under the toll-free policy. The analysis provides an economics-based theoretical foundation for holiday traffic demand management.

The toll-exemption policy deserves particular attention because it is a key institutional mechanism distinguishing Chinese holiday expressway congestion from the holiday traffic growth observed in most other regions. Since 2012, passenger cars with seven seats or fewer have been exempted from expressway tolls during four major statutory holidays, which concentrates price-sensitive private-car demand into a fixed and publicly known time window. The bottleneck analysis of Bao et al. [35] indicates that the resulting concentration is not an incidental by-product but a predictable equilibrium response: departure times cluster around the boundaries of the free period, and part of the associated social cost takes the form of queueing that begins even before the exemption window opens. This mechanism has direct consequences for the methods surveyed in this review, yet it is rarely represented in them. On the prediction side, the models discussed in Section 2 and Section 4 almost never encode the exemption window as an explicit input, even though it induces demand elasticity and structural breaks that violate the stationarity assumptions underlying most architectures; treating policy calendars as first-class covariates, rather than folding holidays into a generic binary indicator, is a concrete and largely unexplored refinement. On the control side, several instruments surveyed in Section 6 change character during the exemption period. Toll-based demand management is unavailable by construction, which removes one of the few demand-side levers and shifts the burden onto ramp metering, variable speed limits, and information guidance. Toll plaza management does not disappear, because vehicles still traverse gantry and lane infrastructure under mixed ETC and MTC configurations, but its objective shifts from transaction processing toward pure throughput and queue management. Transferability should be judged accordingly: findings from settings without comparable exemption policies, such as the Thanksgiving corridor analyses [31], describe holiday demand growth without this institutional amplifier, and methods validated there may understate the abruptness of holiday onsets in the Chinese setting.

These earlier studies and behavioral analyses laid the groundwork for understanding holiday traffic, yet they bear chiefly on the discovery of statistical patterns and on theoretical modeling, with only limited attention to responsive methods. More recently, as big data technologies have matured, researchers have set about constructing more refined holiday prediction methods. Analyzing 441 road segments in Istanbul, Atilgan et al. [36] surfaced the differences between long and short holiday traffic and proposed a holiday-oriented hybrid speed prediction method that marries support vector regression with historical averaging; three feature engineering strategies let the system learn from comparable holidays, and MAPE improved by as much as 29% over holiday periods. Drawing on multi-source data from the Guangdong–Hong Kong–Macao Greater Bay Area, namely weather and traffic volume data at hourly resolution, Lin et al. [37] constructed an integrated prediction model that pairs random effects regression with random forest and accounts for both weather and festival factors. They found Spring Festival volumes to trace a V-shaped curve, and rainfall to act on volume in a nonlinear way, lowering expressway volume on the one side while shifting a portion of trips toward the expressway on the other, a “rain-induced shift” phenomenon.

It is worth noting that traffic emergency response during holiday peak periods faces even more severe challenges than on regular days. Because expressway mainlines and service areas are already operating at or beyond saturated conditions, ground rescue vehicles often cannot reach incident scenes in a timely manner due to congestion, significantly extending incident handling times and readily triggering secondary accidents and widespread congestion propagation. Against this backdrop, the collaborative delivery technology of unmanned aerial vehicles (UAVs) and ground vehicles offers new possibilities for overcoming ground traffic bottlenecks. Wang et al. [38], in their systematic review of UAV–ground vehicle collaborative delivery technologies, noted that traditional ground emergency systems universally suffer from fundamental deficiencies, including slow response speeds, limited coverage, low resource allocation efficiency, and lagging coordination mechanisms. Air–ground integrated collaborative models, by contrast, can significantly enhance emergency response efficiency by leveraging the complementary advantages of ground vehicles’ large payload capacity and long endurance with UAVs’ high speed and strong flexibility.

That study further revealed four major trends in current technological development, namely hybrid optimization algorithms, multi-UAV coordination, artificial intelligence enhancement, and real-time adaptive capability, whose technical characteristics are highly compatible with the rapid response requirements under the dynamic traffic environment of expressways during public holidays. This air–ground collaborative scheduling concept not only holds direct application value for improving the efficiency of accident rescue on expressways during public holidays, but the multi-modal collaborative optimization framework and distributed decision-making architecture involved also provide important methodological reference for constructing a comprehensive emergency management system for expressways during public holidays.

### 4.2. Predicting Holiday Congestion on Expressways

As the principal arteries of holiday travel, expressways pose congestion prediction requirements with characteristics all their own. Using toll data from Jiangsu Province, Chen et al. [39] analyzed in depth how passenger and freight vehicle flows differ across space and time, finding that passenger flow follows a “low from Monday to Thursday, high from Friday to Sunday” rhythm while freight flow runs the other way; over holidays, the inter-city spread in freight volumes narrows markedly relative to working days and weekends. Separating the analysis by vehicle type in this way furnishes an important basis for refining holiday expressway prediction.

For short-term holiday tourism demand, Li et al. [40] developed an improved LSTM model that incorporates spatiotemporal correlations (STC-LSTM), finding that 27.94% of suburban Beijing attractions show a moderate-to-high positive correlation in the way their passenger flows move in step. By folding in auxiliary information, meteorological data, temporal data, and internet search indices, the method gained considerable accuracy over traditional approaches. Aiming at non-stationary traffic flow, holiday and adverse weather scenarios included, Wang et al. [41] proposed the KoopGCN model, which weds Koopman theory to Graph Convolutional Networks, splitting inputs into time-invariant and time-varying components by fast Fourier transform and handling them through dual engine blocks. On expressway monitoring data from Ningde in Fujian Province, the model still predicted accurately even where the training and testing sets differed substantially in distribution.

In the analysis of the traffic volume–travel time relationship on urban expressways, Fujita et al. [42] proposed a continuous one-hour value-based analysis method, using 128 days of minute-by-minute data from the Nagoya expressway network in Japan to plot traffic volume–travel time continuous loop diagrams, enabling not only accurate estimation of daily maximum hourly traffic volumes but also high-accuracy hourly travel time prediction through multiple regression models, providing an intuitive tool for understanding congestion measure effects at the macroscopic multi-hour scale.

For traffic flow prediction on expressway segments under realistic data conditions, Wang et al. [43] proposed the With-Anomaly Detection Probability Attention-BiLSTM (ADP-Attention-BiLSTM) model, which integrates anomaly detection outcomes as an inherent parameter within the prediction framework while incorporating an attention mechanism inside a bidirectional LSTM, thereby addressing limitations in real-time performance and accuracy that arise when anomaly detection results require separate post-processing during data preprocessing. Simulation experiments using actual measured traffic flow data from the Shanghai–Chongqing Expressway demonstrated that the proposed model achieves real-time and accurate short-term traffic flow prediction relative to baseline models. For expressway service area scenarios, Zhao et al. [44] proposed the STL-OMS short-term flow prediction model, which applies the STL algorithm to decompose flow trends and then selects the optimal model for each component.

Table 3 sets these holiday- and expressway-oriented prediction methods side by side. Three technical routes can be read off from it. The first, a “feature engineering plus traditional model” route, makes up for limited model expressiveness through refined extraction of holiday features. The second, a “deep learning plus scenario adaptation” route, strengthens the modeling of non-stationary flow through improved network architectures. The third, a “specialized prediction for critical nodes” route, trains its attention on particular bottlenecks such as toll plazas and peak periods. Table 4 consolidates this comparison, setting the three routes side by side across typical data sources, prediction targets, main advantages, main limitations, and practical applications. Each route has its own emphasis, but a unified framework that would address spatiotemporal heterogeneity, multi-node coordination, and extreme fluctuation all at once has yet to appear. It is also telling that the three routes are validated on different and largely incommensurable datasets, ranging from urban arterials in Istanbul [36] to a provincial toll network [39] and individual expressway corridors [41,43], so no direct evidence exists on which route degrades most gracefully as holiday intensity grows. Establishing shared holiday benchmarks, with consistent definitions of surge onset and severity, would arguably do more to advance this subfield than further architectural variation.

### 4.3. Travel Demand Forecasting and OD Estimation

At bottom, holiday congestion springs from the spatiotemporal imbalance of travel demand. Estimating origin-destination (OD) demand matrices accurately is therefore basic to understanding the causes of congestion at the macroscopic level and to devising control strategies that address those causes at their source. Zhang et al. [45] proposed the Channel-wise Attentive Split-Convolutional Neural Network (CAS-CNN) for urban rail transit, introducing inflow and outflow gating mechanisms to deal with the real-time unavailability of OD data and designing a masked loss function to cope with the high-dimensional sparsity of OD matrices. Tsanakas et al. [46] used floating car data (FCD) to build a data-driven network assignment mechanism, achieving a linear mapping from OD matrices to link flows by capturing congestion effects through the FCD, an approach that sidesteps the complex nonlinear solving that traditional dynamic traffic assignment models demand. Confronting the challenge of online OD prediction for metro systems, Liu et al. [47] proposed the Heterogeneous Information Aggregation Machine (HIAM) module, which makes full use of heterogeneous information, incomplete OD matrices, unfinished order vectors, and DO matrices, to learn the evolution patterns of OD and DO passenger flows jointly.

Beyond conventional traffic data-based OD estimation methods, the integrated utilization of unstructured data opens new possibilities for demand prediction. Rodrigues et al. [48] proposed a deep learning architecture that fuses time series with textual data, combining textual descriptions of activity events with taxi demand time series data through word embeddings, convolutional layers, and attention mechanisms, validating on New York public taxi data that cross-modal information fusion can significantly reduce demand prediction errors in activity areas. The implications for holiday traffic prediction are significant: the massive textual information generated on social media and travel platforms during holidays, including travel guides, trip plans, and traffic condition reports, contains rich travel intention signals, and fusing such data with traditional traffic time series holds promise for compensating for the inherent scarcity of historical holiday samples.

In sum, the analysis of holiday traffic characteristics and the prediction of demand have grown into a multi-level body of research, reaching from the macroscopic discovery of statistical patterns to the microscopic modeling of individual behavior and on to cross-modal data fusion. Notable shortcomings remain, even so. First, studies of holiday traffic characteristics largely stop at statistical description and do not probe deeply into the mechanisms by which congestion arises. Second, the transferability of prediction methods between holiday and regular-day scenarios is weak, most of them requiring ample historical data of the same kind. Third, OD demand estimation has not yet been linked effectively to holiday congestion prediction. How to combine precise demand prediction with deep multi-source fusion so as to achieve network-level congestion situational awareness is the crux that connects prediction technology with control decisions, and the next section takes it up.

## 5. Heterogeneous Data Fusion and Congestion Propagation Analysis

Accurate perception and effective early warning of holiday congestion rest on two foundations: the deep fusion of multi-source heterogeneous data, which supplies comprehensive and reliable information for situational awareness, and a thorough grasp of how congestion propagates and evolves within road networks, which supplies the theoretical basis for judging when and where to warn. The review in this section is organized around these two complementary directions.

### 5.1. Methods for Fusing Heterogeneous Traffic Data

Data sources in modern transportation systems keep growing more varied: loop detectors yield point observations at high temporal resolution, floating car GPS data offers wide spatial coverage but with fluctuating sampling rates, and ETC toll data delivers precise OD information though at a coarser spatial grain. Each source has its strengths and weaknesses in accuracy, coverage, and temporal granularity, and since none on its own can fully characterize network-wide traffic states, multi-source fusion becomes unavoidable.

The Macroscopic Fundamental Diagram (MFD), a powerful means of describing the traffic state of an urban road network, depends heavily on data quality for its estimation to be accurate. Ambühl and Menendez [49] did foundational work here, proposing an algorithmic framework that splits the network into detector-covered and uncovered sub-networks and uses loop data and floating car data, respectively, for a fused estimate; simulations on the Zurich network confirmed that the fusion consistently and substantially lowers MFD estimation error, even when the floating car penetration rate has to be inferred from loop detectors and is spread unevenly across space. Saffari et al. [50] carried MFD fusion estimation further into large-scale network settings with a Bayesian fusion framework that brings together floating car data of unknown penetration rate and near-full-sample loop-based data, improving average density estimation especially markedly and reconfirming the familiar limitation that loop detectors cannot measure segment-level average density accurately.

In expressway scenarios, the fusion of heterogeneous sensor data is critical for improving traffic state estimation accuracy. Zhang et al. [51] proposed a data-driven optimization method for expressway traffic state estimation that fuses the broad coverage advantage of ETC data with the fine-grained advantage of detector data, using probabilistic methods to capture interdependencies between segment traffic states and their upstream/downstream neighbors, achieving mean absolute percentage errors of only 0.9% to 2.3% during peak periods on field data from the G92 expressway in Zhejiang Province. In a complementary vein, Chen et al. [52] proposed the Adaptive Rolling Smoothing (ARS) method, which tunes filter parameters dynamically to characterize in fine grain how congestion forms, propagates, and dissipates; by fusing aggregate measurements from remote microwave sensors with individual vehicle timestamps from license plate recognition cameras, it reconstructs spatiotemporal speed maps effectively at weaving, merging, and diverging segments.

**Figure 2:**
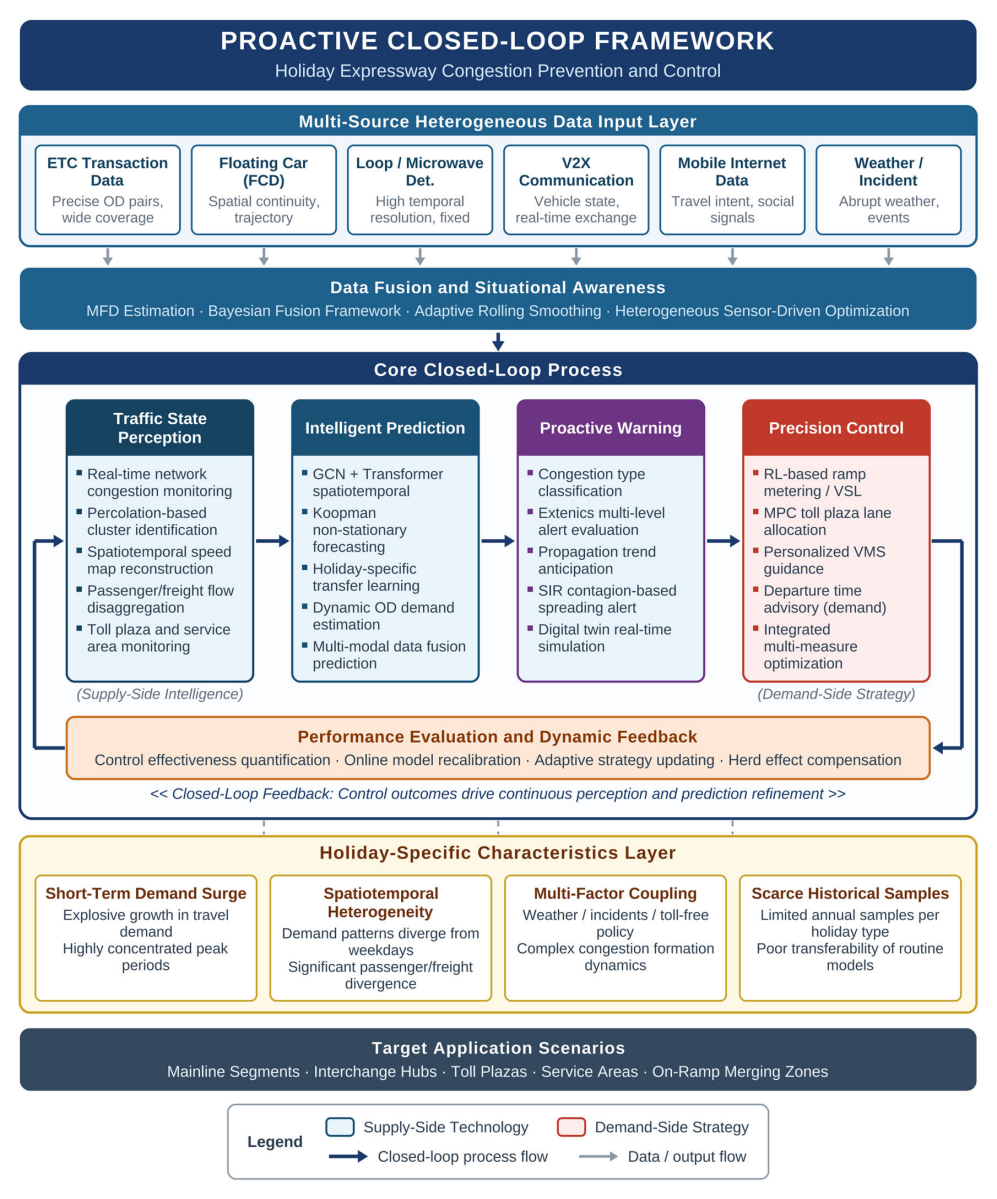
Closed-loop system framework for proactive congestion prevention and control for expressways during public holidays.

From the perspective of this review’s overall framework, the fusion methods surveyed above constitute the perception layer on which every downstream stage depends, and it is worth making the sensing basis explicit. The sensor portfolio of a modern expressway spans fixed point detectors, including inductive loops and remote microwave sensors, which provide high-frequency flow, speed, and occupancy observations at sparse locations [52]; license plate recognition cameras, which yield individual vehicle re-identification between gantries [52]; ETC gantry transactions, which offer near-complete samples of vehicle passages with precise timestamps and origin-destination structure [51]; floating car and GPS trajectory data, which contribute spatially continuous but penetration-dependent speed observations [11,20,49,50]; and video-based computer vision, which supports incident and queue detection [21]. Each modality degrades differently under holiday conditions. Fixed detectors lose discriminating power when flow approaches capacity across most of the network at once; floating car penetration and composition change as the vehicle mix shifts from commuting fleets toward private cars; and the composition of toll-lane records can shift when exemption periods alter lane choice and processing procedures. Holiday-oriented perception therefore requires fusion architectures that are robust not merely to random missing data but to systematic, scenario-correlated shifts in the reliability of each source, a stronger requirement than the ones addressed in most of the fusion literature reviewed here [49,50,51]. In the closed-loop framework of Section 7.2 and Figure 2, this layer supplies the real-time state estimates that initialize prediction, the observations against which warnings are verified, and the measurements from which control effectiveness is evaluated, so its holiday robustness sets the performance ceiling of the entire loop.

### 5.2. Network-Level Analysis of Congestion Propagation Mechanisms

Understanding how congestion propagates through a road network matters decisively for forward-looking early warning. Only when one knows “where congestion comes from and where it is headed” can targeted interventions be applied before it spreads widely. Of late, percolation theory has furnished a powerful theoretical instrument for analyzing congestion propagation at the network level.

Hamedmoghadam et al. [53] proposed a percolation analysis framework grounded in flow heterogeneity, one that can identify in theory the bottleneck segments with a decisive bearing on network flow transmission; on large-scale real networks, they showed that relieving congestion on just a few such segments yields significant gains in network performance. The same team went on to join percolation theory with dynamic boundary control [54], proposing a dynamic perimeter control method that reshapes the control boundary as congestion advances, keeping small congested zones from coalescing into large congestion clusters and offering a fresh route to proactive intervention.

From a dynamical modeling perspective, Saberi et al. [55] creatively applied epidemic spreading models to urban traffic congestion analysis, analogizing the spreading and dissipation of congestion within road networks to the classical SIR (Susceptible–Infected–Recovered) model, proposing two macroscopic characteristic parameters, namely congestion spreading rate β and congestion recovery rate μ, and validating the effectiveness of this analogy through multi-city empirical analysis. This study demonstrates that the propagation of traffic congestion can essentially be described by a simple contagion process, providing not only a parsimonious yet powerful theoretical framework for monitoring and predicting the temporal evolution of the proportion of congested segments in road networks but also offering an analogical approach for blocking congestion propagation through “quarantine” strategies.

At a higher theoretical plane, Ambühl et al. [56] integrated percolation theory with the MFD organically and uncovered a key result: the instant at which the number of congestion clusters in the network peaks coincides exactly with the instant at which MFD flow reaches its maximum, beyond which the network risks percolation, that is, traffic breakdown. This insight draws a parallel between the network-average flow’s shift from the uncongested to the congested phase and the percolation transition from scattered congestion to large-scale connectivity, opening an entirely new vantage point on network resilience and the mechanisms of congestion breakdown.

Multi-source data fusion confers comprehensive perception of network-wide traffic states, while the theory of congestion propagation lays bare the patterns that govern how congestion evolves. Brought together, the two complete the technical chain of perception, forecasting, and early warning for a holiday congestion management system. The ultimate point of early warning, however, is to drive effective control. Turning the perceived congestion situation and the predicted propagation trend into concrete control measures and traveler guidance strategies, and closing effective feedback loops as these are implemented, is the “last mile” from prediction and warning to proactive prevention and control, which is what the next section addresses.

## 6. Traffic Control and Traveler Guidance on Expressways

The fundamental worth of congestion prediction, forecasting, and early warning technologies lies in their driving effective control. For managing expressway congestion over public holidays, this plays out along two intertwined dimensions: on the supply side, the proactive control measures of traffic authorities, among them variable speed limits, ramp metering, and toll plaza management; and on the demand side, information guidance directed at travelers, which steers the spatiotemporal distribution of demand by shaping travelers’ choices of time and route. This section reviews the field from the standpoints of expressway control technologies and traveler behavior guidance.

### 6.1. Expressway Active Traffic Control Technologies

Expressway operations management contends with challenges both routine and sudden, and effective control technologies are vital to keeping traffic efficient. Wang et al. [57] drew on ETC data to study both the long-term and the short-term effects of COVID-19 traffic restriction policies on expressway volumes in Zhejiang Province, characterizing the temporal profile of those effects with the Prophet model together with the notion of traffic volume loss, and bringing out the relationship between case counts and policy effects through Granger causality tests and Bayesian vector autoregression models, all of which offers a methodological reference for assessing traffic impacts under comparable emergencies.

On ramp metering, Han et al. [58] proposed a physics-informed reinforcement learning ramp control strategy that trains the reinforcement learning model on a blend of historical data and synthetic data generated by traffic flow models, with the iterative training evaluating control strategies in the real environment rather than in simulators, thereby escaping the way conventional reinforcement learning is hemmed in by inaccurate training environments. The strategy, by the experimental results, outperforms classical feedback-based ramp control significantly in total travel time saved. This reinforcement learning approach to ramp control mirrors a broader shift in traffic control, away from fixed rules and toward adaptive learning. Wang et al. [59], in a perspective review of multi-intersection cooperative control platform frameworks for connected and automated vehicle environments, traced the same evolution from fixed-time and actuated control toward intelligent data-driven approaches such as deep reinforcement learning, and identified the persistent gap between strong simulation performance and reliable operation under complex, uncertain field conditions, together with the cold-start instability of such controllers during initial deployment, as central obstacles to engineering application. To address these obstacles, that review advocated a decoupled platform architecture in which data management and algorithm integration evolve independently, allowing learning-based control strategies to be updated and reconfigured without interrupting operation, and grounded network-wide perception in the fusion of loop detector, video, GPS, and ETC data rather than any single source whose coverage or reliability may fluctuate. Reinforcement learning, as a closed-loop feedback adaptive control paradigm that requires only system input–output interaction data without precise mathematical models of the controlled object, possesses favorable compatibility with both existing detection facilities and emerging Vehicle-to-Everything (V2X) communication technologies. This characteristic holds direct relevance for expressway management: core principles such as model-free self-learning and real-time strategy updating can be directly transferred to adaptive control of expressway ramps, toll plazas, and mainline segments, especially during holiday periods when these scenarios exhibit complex nonlinear time-varying characteristics that are difficult to precisely pre-model.

In the coordinated optimization of variable speed limits and ramp metering, Carlson et al. [60] proposed a systematic approach that incorporates both control measures within a unified discrete-time optimal control framework, quantifying the impact of variable speed limits on traffic flow through a macroscopic second-order traffic flow model and efficiently solving constrained optimization problems for large-scale networks using the feasible direction algorithm. A key finding is that the integrated application of variable speed limits with coordinated ramp control achieves significant traffic efficiency improvements compared with either measure alone, providing a theoretical basis for multi-measure coordinated control during holiday periods.

Incident detection and safety management are equally core aspects of expressway management. Liu et al. [61] proposed a Bayesian deep learning method for expressway incident detection that quantifies the uncertainty of its results by modeling the aleatoric uncertainty of the data and the epistemic uncertainty of the model jointly, surpassing baseline methods on detection accuracy, detection rate, and false alarm rate alike. Li et al. [62] addressed two inherent challenges in incident detection, namely small sample sizes and class imbalance, by proposing a hybrid model integrating Generative Adversarial Networks (GAN) with Temporal–Spatial Stacked Autoencoders (TSSAE), augmenting and balancing incident samples through GAN before utilizing TSSAE to simultaneously extract spatiotemporal correlation features of traffic flow for incident detection. For mixed traffic flow scenarios in which human-driven and autonomous vehicles coexist, Jin et al. [63] proposed a two-stage Variable Speed Limit (VSL) modeling framework: the first stage carries out macroscopic VSL optimal control on an extended cell transmission model, and the second validates the strategy through VISSIM microsimulation, with the experiments showing gains in both traffic efficiency and safety.

Turning to toll plaza management at this critical expressway node, Zhou et al. [64] designed a proactive control method based on model predictive control that wields two instruments: dynamic lane configuration (adjusting the numbers of ETC and MTC lanes on the fly) and variable speed limits. The method predicts short-term demand with LSTM, models the evolution of traffic around the plaza with the cell transmission model, and minimizes total vehicle travel time within a rolling optimization framework. VISSIM microsimulation confirmed that the method improves toll plaza performance substantially, providing a systematic answer to the characteristic problem of holiday toll plaza congestion.

### 6.2. Information Services and Traveler Guidance

Travelers’ route choice behavior is the bridge that links traffic demand to network loading. By disseminating information in a targeted way to prompt travelers toward sensible adjustments of their plans, the spatiotemporal distribution of demand can be reshaped without adding infrastructure, which makes this an important demand-side measure against holiday congestion.

Variable Message Signs (VMS), the most mature of roadside information dissemination facilities, have drawn fairly systematic study of how they influence driver behavior. From a stated preference survey of 9600 drivers in Beijing, Ma et al. [65] built a multinomial logit model of driver response under VMS information, finding gender, the type of vehicle driven, familiarity with alternative routes, and the usefulness and comprehensibility of the VMS information all significantly associated with diversion behavior, whereas age was not. Zhao et al. [66] went further into driver preferences for the content and format of VMS through combined revealed preference and stated preference surveys, finding that under foggy conditions drivers favor amber text, a graphic format of blue text on a white background, or single-line suggested diversion information, with preferences differing significantly by weather condition, education level, and trip purpose.

Notably, early empirical studies on the influence of traffic information on route choice laid important foundations for subsequent work. Emmerink et al. [67], based on large-scale traveler survey data from the Amsterdam corridor, used binary ordered probit and other discrete choice models to find that the influence mechanisms of broadcast traffic information and VMS information on route choice are highly similar and positively correlated, commuters are less influenced by traffic information than non-commuters, and male drivers on business trips have a higher willingness to pay for in-vehicle dynamic traffic information. These early findings established the fundamental understanding that information effectiveness depends on traveler characteristics and travel context, upon which subsequent research has further deepened and expanded.

Route choice behavior research has further revealed the context-dependent nature of VMS information effectiveness. Drawing on empirical data from Madrid, Romero et al. [68] studied how VMS affects diversion between toll highways and free alternative routes, finding that incident information paired with travel time estimates produces the largest rise in diversion toward the toll route, and that the effectiveness of the information varies with the type of day. Zhao et al. [69] used the CHAID decision tree algorithm to sort drivers into five groups by their characteristics and, through random parameter logistic regression models, brought out the within-group heterogeneity of VMS information effectiveness. Incident information, the results show, sways route choice more than construction or congestion information does; drivers with more than six years of experience respond more to quantitative delay information, while less experienced female drivers respond more to display format and frequency. Underpinning effective traveler guidance is the capacity of the sensing infrastructure to provide accurate and timely traffic state information. Luo et al. [70], in their review of ETC-enabled intelligent expressway technologies, demonstrated that fusing ETC gantry transaction data with video, radar, and GPS sources substantially improves traffic state estimation for expressway corridors, and argued that establishing a sensing, fusion, decision, and control closed-loop pipeline anchored on ETC gantry infrastructure constitutes a key direction for intelligent expressway transformation. This perception infrastructure perspective is particularly relevant for holiday congestion management, as the quality of information disseminated through VMS and navigation platforms directly determines whether guidance achieves beneficial demand redistribution or instead triggers collective overreaction.

The group heterogeneity documented above points to a question that holiday congestion management has largely left implicit: management measures do not distribute their benefits and burdens evenly across traveler groups. Information-based guidance chiefly benefits travelers who receive, trust, and can act on the information, which in practice favors experienced drivers and travelers familiar with alternative routes [65,69] and depends on the quality and timeliness of the disseminated information [70]; unfamiliar long-distance holiday travelers, precisely the group that grows most during holidays, are less able to exploit diversion advice. Ramp metering protects mainline throughput at the cost of queueing on entering ramps, shifting delay from long-distance through traffic toward local and short-distance users. Reconfiguring toll plaza lanes between ETC and MTC alters relative waiting times across payment-type groups [64], and the toll-exemption policy itself benefits eligible passenger cars while freight and coach operators absorb part of the resulting congestion externality [35,39]. These distributional consequences matter in two ways. Practically, perceived unfairness undermines compliance with guidance, and compliance is the mechanism through which demand-side measures take effect at all [68]. Analytically, almost none of the control studies surveyed in this section report group-differentiated outcomes, so the equity profile of holiday congestion management remains an open empirical question. Future evaluations should report impacts disaggregated by trip purpose, trip length, route familiarity, and vehicle type rather than network averages alone.

Information guidance, however, does not always work to good effect. From the standpoint of stochastic user equilibrium theory, Zhou et al. [71] explored at depth how travelers’ herding effects bear on route choice, showing that building herding into the stochastic user equilibrium model, where route flow information is available, can resolve three matters left unsettled in the literature: the stability of SUE, the existence of meaningful link tolls that steer SUE toward the system optimum, and the design of trial-and-error congestion pricing under non-equilibrium observed flows. Its central finding, that herding can offset travelers’ perception errors, carries weighty policy implications for the design of navigation software and for congestion pricing strategies.

To sum up, active expressway traffic control already spans a range of measures, ramp metering, variable speed limits, and dynamic toll plaza management among them, while research on traveler behavior guidance has amassed a rich understanding of VMS information effectiveness. A notable gap remains, though, in the disconnect between control measures and prediction systems: the outputs of prediction models often do not drive the dynamic adjustment of control strategies directly, and the results of control are not fed back to the prediction systems for real-time calibration. In holiday scenarios in particular, the high behavioral synchronization among travelers means that information guidance may set off feedback effects such as secondary congestion, which makes urgent the construction of a closed-loop control system that integrates prediction, guidance, feedback, and iterative re-prediction.

## 7. Conclusions and Future Prospects

### 7.1. Summary of Findings and Limitations

Around the core theme of predicting and proactively managing expressway congestion during public holidays, this paper has reviewed research progress along five dimensions and has distilled from each the gap that motivates the framework developed in Section 7.2. In traffic speed and flow prediction (Section 2), methods have advanced from statistical models to graph-based and Transformer-based spatiotemporal architectures, yet they remain calibrated to regular patterns and largely untested against holiday non-stationarity. In congestion identification and early warning (Section 3), the chain from prediction to graded alerting has taken shape, but classification standards presuppose weekday baselines and lack a management-oriented, calendar-conditioned formulation. In holiday characteristics and demand analysis (Section 4), statistical and behavioral regularities are well documented and the toll-exemption mechanism is theoretically understood, yet holiday-specific prediction remains fragmented across incompatible datasets and OD estimation is not yet coupled to congestion forecasting. In data fusion and propagation analysis (Section 5), MFD-based fusion and percolation theory provide network-level perception and breakdown analysis, but their validation in holiday settings remains thin. In control and guidance (Section 6), a mature toolbox of supply-side and demand-side instruments exists, but it operates open-loop with respect to prediction, and behavioral feedback such as herding is not designed for. These five gaps are complementary rather than parallel: each corresponds to one stage of the closed-loop system developed in the next subsection.

Three limitations of this review should also be acknowledged. First, as a structured narrative review rather than a PRISMA systematic review, it does not report itemized record counts, and the corpus was assembled with an emphasis on methodological representativeness rather than exhaustive enumeration. Second, holiday-specific evidence remains concentrated in the Chinese institutional context, so findings tied to the toll-exemption mechanism may transfer only partially to regions with different holiday structures and toll policies; a systematic cross-regional comparison of holiday traffic management remains a valuable task for future work. Third, the quantitative performance figures quoted from primary studies were obtained on heterogeneous datasets under differing definitions and are therefore not directly comparable across studies.

### 7.2. Priorities for Future Research

Based on the review analysis across the above five dimensions, this paper proposes a closed-loop system framework encompassing perception, prediction, warning, control, and feedback for proactive congestion prevention and control for expressways during public holidays, as illustrated in Figure 2. This framework is founded on multi-source heterogeneous data fusion, with real-time traffic state perception, intelligent prediction, proactive alerting, and precision control as core processes, and dynamic evaluation of control effectiveness and online model calibration as feedback mechanisms, forming a continuously optimizing closed-loop system. The framework’s foundation comprehensively accounts for four distinctive characteristics of holiday traffic, namely short-duration surges, spatiotemporal heterogeneity, multi-factor coupling effects, and historical sample scarcity, as constraint conditions for technical solution design at each stage, ultimately providing systematic proactive congestion prevention and control capabilities for typical application scenarios including expressway mainline segments, interchange hubs, toll plazas, service areas, and ramp merging areas.

The framework is grounded directly in the five review dimensions, and the correspondence can be stated explicitly. The perception stage builds on the multi-source fusion and sensing methods of Section 5.1, extended by the trajectory-based estimation techniques discussed in Section 2.4 [20,49,50,51,52,70]. The prediction stage draws on the speed and flow prediction architectures of Section 2 together with the holiday-specific and demand-oriented models of Section 4 [16,36,37,41,45]. The warning stage corresponds to the congestion identification, classification, and graded early warning methods of Section 3 [23,28,29,30]. The control stage integrates the supply-side instruments and demand-side guidance of Section 6, including ramp metering, variable speed limits, toll plaza management, and information dissemination [58,60,63,64,65,68]. The feedback stage operationalizes two findings that recur across the review: control effects must be measured through the same perception layer that initializes prediction [51,70], and guidance-induced behavioral responses, herding among them [71], must be fed back into the next prediction cycle rather than treated as exogenous noise.

It is fair to ask what this framework adds relative to established reference architectures. Conventional Advanced Traffic Management Systems (ATMS) already implement a sense, decide, and act cycle, and decision support systems built on dynamic traffic assignment (DTA) already couple demand models to network loading. The framework proposed here differs from both in three specific ways rather than in its general shape. First, typical ATMS deployments are predominantly reactive: control plans are triggered by detected states, and prediction, where present, serves traveler information displays rather than actuation. In the proposed loop, prediction outputs directly drive warning grades and control activation, with the four holiday characteristics imposed as explicit design constraints at every stage. Second, DTA-based architectures presuppose calibrated demand and supply models and equilibrium-like behavior, assumptions that holiday conditions violate through demand surges, policy-induced departure clustering [35], and information-driven herding [71]; the proposed loop therefore replaces one-shot equilibrium assignment with rolling prediction and online recalibration, in the spirit of the model predictive control scheme of [64] but extended from a single toll plaza toward the corridor scale. Third, neither reference architecture treats the path from control effect evaluation back to model recalibration as a first-class component, whereas here it is the mechanism that compensates for holiday sample scarcity, because each holiday deployment generates exactly the scarce observations that the next prediction cycle lacks. The framework should therefore be read not as a replacement for ATMS or DTA infrastructure but as a holiday-specialized operating discipline layered on top of it.

A concrete walk-through illustrates the intended operation. Consider an outbound expressway corridor on the morning of the first day of a major holiday. In the perception stage, ETC gantry transactions fused with detector and floating car data [49,51] establish segment states and reveal a demand wave forming earlier than the historical holiday average. In the prediction stage, a holiday-adapted model conditioned on the toll-exemption calendar and the weather forecast [37,41] projects that two interchange merge areas will break down within the next 40 to 60 min. In the warning stage, the projected states are mapped to graded alerts using classification logic of the kind surveyed in Section 3 [30], and the surge is distinguished from incident-induced congestion so that the appropriate response type is selected. In the control stage, ramp metering is activated at the two threatened merges [58], variable speed limits smooth the approaching flow [60,63], toll plaza lane configurations are shifted toward maximum throughput [64], and variable message signs together with navigation platforms disseminate staged diversion guidance formulated to avoid synchronized rerouting [68,71]. In the feedback stage, the perception layer measures realized speeds and diversion rates, the deviation between predicted and realized breakdown times recalibrates the prediction model online, and observed guidance compliance updates the behavioral parameters used in the next cycle. Each stage consumes the output of the previous stage and produces a measurable intermediate product, which is what distinguishes a closed loop from a pipeline of independently optimized modules.

Based on this framework, this paper identifies the following priority directions for future research:

(1) Building holiday-specific prediction models. General-purpose models struggle to accommodate the distinctive holiday pattern of short-duration surges and pronounced spatiotemporal heterogeneity. Future work should develop specialized models able to identify and handle non-stationary flow adaptively. Promising avenues include time-varying dynamics modeling that draws on Koopman theory, cross-holiday knowledge transfer built on transfer learning, and multi-modal prediction frameworks that fold in weather, events, and policy factors. Pre-trained spatiotemporal foundation models offer a further avenue: large models pre-trained on traffic data from many networks and periods could be fine-tuned on the scarce holiday samples of a target corridor, easing the sample-scarcity constraint that limits holiday-specific training.

(2) Refined identification of congestion types and differentiated control. There is a pressing need for unified standards and identification systems for congestion types that mark out clearly the feature boundaries, propagation mechanisms, and optimal responses for different types, localized standstill, widespread slow-moving, and incident-induced among them, so as to give differentiated control a scientific footing.

(3) Building a closed-loop system that integrates prediction, warning, control, and feedback. Linking the whole pipeline, from the outputs of prediction models through the formulation of control strategies to feedback on their effectiveness, is the core challenge in attaining genuine proactive management. Future work should seek ways to integrate technologies such as reinforcement learning and digital twins deeply with traffic control, so as to build dynamic closed-loop systems that update strategies in real time.

(4) Deep fusion and real-time perception of multi-source heterogeneous data. As ETC systems, cooperative vehicle–infrastructure equipment, and mobile internet data advance rapidly, achieving real-time fusion, anomaly detection, and situational prediction of multi-source data in holiday scenarios is critical to making early warning more timely. Robust fusion algorithms that cope with missing data, delay, and noise need to be developed.

(5) Precise modeling of traveler behavior and personalized guidance. Backed by big data and artificial intelligence, future work should investigate in depth how different traveler groups respond, develop differentiated dissemination strategies keyed to individual profiles, and account fully for the feedback between information guidance and traffic flow, so as to avoid the secondary concentration of demand that dissemination can cause. Equity deserves explicit treatment within this direction: guidance and control strategies should be evaluated not only on network efficiency but also on how their costs and benefits are distributed across traveler groups, so that holiday management does not systematically disadvantage less informed or less flexible travelers.

(6) System integration and validation for practical deployment. Current research is validated for the most part in simulation environments or on offline data. Future efforts should strengthen integration validation and the translation to application aimed at real traffic management systems, conducting large-scale empirical studies in densely networked regions in particular, so as to carry research outcomes from academic exploration toward engineering implementation.

## Figures and Tables

**Table 1:** Comparison of representative spatiotemporal traffic speed/flow prediction models.

Model	Ref.	Spatial	Temporal	Key Innovation	Data
Deep Learning	[5]	Linear + tanh	N/A	Special event flow capture	Chicago I-55
Attn-Conv-LSTM	[8]	CNN	Attn-LSTM + Bi-LSTM	Attention weighting + dual periods	Traffic flow
GCN-GAN	[10]	Improved GCN	N/A	Generative adversarial framework	Urban network
Graph CNN-LSTM	[11]	Graph CNN	LSTM	GPS trajectory + dim. reduction	Xi’an/Chengdu DiDi
GATCN	[14]	Graph attention	Temporal conv.	Multi-head self-attention coupling	Expressway
STGI-ResNet	[12]	Markov graph conv.	Residual + Inception	Linear complexity for large networks	Large-scale urban network
MD-GCN	[13]	Dual graph conv. (GraphSAGE + MGCN)	Multi-scale gated conv.	Local + global node correlation	Multiple public datasets
AGC-Seq2Seq	[15]	Graph conv.	Seq2Seq + attention	Multi-step error accumulation mitigation	Real network

**Table 2:** Classification and comparison of congestion identification and early warning methods.

Method Category	Ref.	Core Technology	Target	Key Contribution
Periodic conv. pred.	[23]	PCNN multi-granularity	Short-term congestion	Time series folding + multi-scale modeling
Connected vehicle	[24]	ML + trajectories	State (97% acc.)	Micro disturbance to macro congestion mapping
Spatiotemporal joint	[25]	Conv-BiLSTM	Spatiotemporal state	CNN spatial + BiLSTM temporal fusion
Vision Transformer	[26]	ViT + CNN + LSTM	Urban congestion	Superior in anomalous scenarios
Weather-influenced	[27]	LSTM/AR-LSTM/CNN	Road flow under weather	Meteorological data fusion; CNN best
Comprehensive class.	[28]	Improved FCM	Multi-class traffic state	Novel metric + membership improvement
Multi-view GNN	[29]	MvFCGCN	Expressway state	Multi-source fusion + deep clustering
Extension warning	[30]	VMD-ELM + extension	Level (>97% acc.)	Prediction to warning full pipeline

**Table 3:** Comparison of traffic prediction methods for holiday and expressway scenarios.

Study	Prediction Target	Core Method	Data Source	Holiday/Special Scenario Strategy
[36]	Holiday speed	SVR + HA hybrid	Istanbul segments	Long/short holiday classification + feature engineering
[37]	Hourly volume	RER + RF fusion	GBA multi-source	Weather × festival interaction modeling
[39]	Passenger/freight flow	Toll-data spatiotemporal differentiation analysis	Jiangsu toll data	Vehicle-type spatiotemporal differentiation calendar
[40]	Tourism demand	STC-LSTM	Beijing attractions	Inter-attraction spatial correlation + auxiliary info
[41]	Non-stationary flow	KoopGCN	Fujian expressway	Koopman decomposition of time-varying components
[43]	Expressway segment flow	ADP-Attention-BiLSTM	Shanghai–Chongqing Expy	Anomaly detection probability integration
[42]	Volume–travel time	Continuous 1-hr + reg.	Nagoya expressway	Congestion loop diagram + macro analysis

**Table 4:** Comparison of the three technical routes for holiday expressway traffic prediction.

Technical Route (Refs.)	Typical Data Sources	Prediction Targets	Main Advantages	Main Limitations	Practical Applications
Feature engineering + classical models [36,37,39]	Segment speeds; hourly volume with weather; toll records	Holiday speed; hourly volume; vehicle-type flows	Interpretable; low data and computing demand; exploits comparable-holiday similarity	Limited capacity for abrupt nonlinear surges; manual feature design	Corridor-level forecasting and traveler information
Deep learning + scenario adaptation [40,41,43]	Attraction flows with search indices; expressway monitoring data; section flow	Tourism demand; non-stationary flow; section flow	Strong nonlinear spatiotemporal capacity; robust to distribution shift; fuses auxiliary signals	Data- and computation-intensive; scarce holiday samples; limited interpretability	Network- and segment-level prediction under holiday and adverse weather
Specialized prediction for critical nodes [42,44]	Minute-level urban expressway data; service area flow	Volume–travel time relation; service area flow	Matches bottleneck dynamics; supports node-level operations directly	Node-specific scope; cross-node transferability untested	Service areas, peak periods, and bottleneck management

## Data Availability

No new data were created or analyzed in this study. Data sharing is not applicable to this article.

## Abbreviations

The following abbreviations are used in this manuscript:

ADP-Attention-BiLSTM	Anomaly Detection Probability Attention Bidirectional Long Short-Term Memory
AGC-Seq2Seq	Attention Graph Convolution Sequence-to-Sequence
ARIMA	Autoregressive Integrated Moving Average
ARS	Adaptive Rolling Smoothing
BiLSTM	Bidirectional Long Short-Term Memory
CAS-CNN	Channel-wise Attentive Split Convolutional Neural Network
CHAID	Chi-squared Automatic Interaction Detector
CNN	Convolutional Neural Network
Conv-BiLSTM	Convolutional Bidirectional Long Short-Term Memory
DCNN	Deep Convolutional Neural Network
DO	Destination-Origin
ELM	Extreme Learning Machine
EM	Expectation-Maximization
ETC	Electronic Toll Collection
FCD	Floating Car Data
FCM	Fuzzy C-Means
GAN	Generative Adversarial Network
GATCN	Graph Attention Temporal Convolutional Network
GCN	Graph Convolutional Network
GNN	Graph Neural Network
GPS	Global Positioning System
GraphSAGE	Graph Sampling and Aggregation
HA	Historical Average
HIAM	Heterogeneous Information Aggregation Machine
KNN	k-Nearest Neighbor
KoopGCN	Koopman Graph Convolutional Network
LOESS	Locally Estimated Scatterplot Smoothing
LSTM	Long Short-Term Memory
MAPE	Mean Absolute Percentage Error
MD-GCN	Multi-scale Temporal Dual Graph Convolution Network
MFD	Macroscopic Fundamental Diagram
MGCN	Mix-hop Propagation Graph Convolution
ML	Machine Learning
MTC	Manual Toll Collection
MvFCGCN	Multi-view Fusion Chebyshev Graph Convolutional Network
OD	Origin-Destination
PCNN	Periodic Convolutional Neural Network
RER	Random Effects Regression
RF	Random Forest
Seq2Seq	Sequence-to-Sequence
SIR	Susceptible-Infected-Recovered
SRCN	Spatiotemporal Recurrent Convolutional Network
ST	Spatiotemporal
STC-LSTM	Spatiotemporal Correlation Long Short-Term Memory
STGI-ResNet	Spatiotemporal Graph Inception Residual Network
STL	Seasonal-Trend Decomposition Using LOESS
STL-OMS	Seasonal-Trend Decomposition Using LOESS with Optimal Model Selection
SUE	Stochastic User Equilibrium
SVM	Support Vector Machine
SVR	Support Vector Regression
TFT	Temporal Fusion Transformer
TSA-SL	Time Series Analysis with Supervised Learning
TSSAE	Temporal–Spatial Stacked Autoencoder
UAV	Unmanned Aerial Vehicle
V2X	Vehicle-to-Everything
VAR	Vector Autoregression
ViT	Vision Transformer
VMD	Variational Mode Decomposition
VMS	Variable Message Sign
VSL	Variable Speed Limit
